# Common origin of methylenedioxy ring degradation and demethylation in bacteria

**DOI:** 10.1038/s41598-017-07370-x

**Published:** 2017-08-07

**Authors:** Hisashi Takeda, Kazuki Ishikawa, Hinaka Yoshida, Daisuke Kasai, Daigo Wakana, Masao Fukuda, Fumihiko Sato, Tomoo Hosoe

**Affiliations:** 1 Department of Organic Chemistry, Hoshi University, Ebara, Shinagawa Tokyo, Japan; 2 Department of Bioengineering, Nagaoka University of Technology, Kamitomioka, Nagaoka, Niigata Japan; 3 Division of Integrated Life Science, Graduate School of Biostudies, Kyoto University, Kitashirakawa, Sakyo, Kyoto Japan

## Abstract

Plants produce many specific secondary metabolites as a response to environmental stress, especially biological stress. These compounds show strong biological activities and high stability against degradation by microbes and animals. Berberine, a benzylisoquinoline alkaloid, is found in many plant species and has strong antimicrobial activity, and is often included in traditional herbal medicines. We previously investigated how berberine is degraded in nature and we isolated two berberine-utilizing bacteria. In this study, we characterized the gene encoding the enzyme that degrades the 2,3-methylenedioxy ring of berberine; this ring is important for its activity and stability. Further characterization of several other berberine-utilizing bacteria and the genes encoding key demethylenation enzymes revealed that these enzymes are tetrahydrofolate dependent and similar to demethylation enzymes such as GcvT. Because the degradation of *O*-methyl groups or the methylenedioxy ring in phenolic compounds such as lignin, lignan and many other natural products, including berberine, is the key step for the catabolism of these compounds, our discovery reveals the common origin of the catabolism of these stable chemicals in bacteria.

## Introduction

Plants produce many secondary metabolites to respond to environmental stress, especially biological stress. These compounds have strong biological activities and high stability against degradation by microbes and animals. Berberine (BBR) is a benzylisoquinoline alkaloid that is produced by various higher plants, such as *Coptis japonica*, *Berberis fremontii* and *Mahonia aquifolium* ^1^, and shows strong antimicrobial and chemical defense activities^2^. BBR was also used as an antidiarrheal in ancient times, and interest in its use toward improving intestinal microflora is increasing^3, 4^. Although few studies have characterized the structure-activity relationship of protoberberine alkaloids, chemicals with a methylenedioxy ring, such as BBR, tend to demonstrate more activity than those with dimethoxy or hydroxy groups, such as palmatine^5, 6^.

The metabolism of BBR has been mainly investigated in mammals. CYP2D6, CYP1A2 and CYP3A4 are responsible for metabolizing BBR to demethyleneberberine (D-BBR) in human liver microsomes^7^. Interestingly, BBR was not metabolized by the intestinal bacteria of humans and rats in anaerobic conditions^8, 9^. Liquid

chromatography/time-of-flight mass spectrometry analysis of bile, plasma and urine in rats revealed the presence of BBR metabolites, including thalifendine, berberrubine, D-BBR, jatrorrhizine, palmatine, columbamine, 3,9-demethyl-palmatine, hydroxylated BBR and hydroxylated D-BBR; BBR was converted into sulfate and glucuronic acid conjugates for excretion^10^.

**Figure 1:**
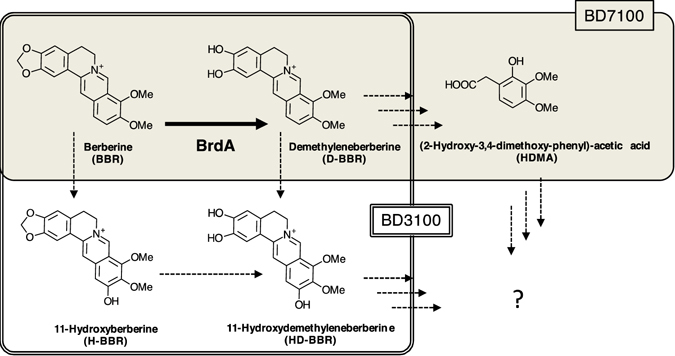
The BBR metabolites and deduced degradation pathway in BD3100 and BD7100. The BBR metabolites identified in BD7100 and BD3100 are boxed by a double line (with a filled background) and a single line, respectively. The thick arrow indicates demethylenation at the 2,3-methylenedioxy ring of BBR, which was a focus of this study. The dotted arrows indicate the deduced degradation pathway.

There is currently a new opportunity to study the environmental fate of BBR: two bacteria, *Sphingobium* sp. strain BD3100 and *Rhodococcus* sp. strain BD7100, that utilize BBR as their sole carbon source were isolated from the sludge of a berberine-producing factory^11^. The formation of demethyleneberberine (D-BBR), a common metabolite of BBR, was detected in both strains, whereas 11-hydroxyberberine (H-BBR) and 11-hydroxydemethyleneberberine (HD-BBR) were unique to BD3100^11^, and 2-hydroxy-3,4-dimethoxybenzeneacetic acid (HDBA) was unique to BD7100^12^. This result suggests that demethylation is the key step in the catabolism of BBR in these bacteria (Fig. 1).

To isolate the BBR-demethylenation enzyme from the bacteria, we used a transposon tagging method; the function of a gene, designated *brdA*, was confirmed using recombinant protein that was produced in *E. coli*. Furthermore, other BBR-degraders, *Arthrobacter* sp. strain GBD-1 and *Burkholderia* sp. strain CJ1, were isolated, and their BrdA homologs were characterized. All BrdA homologs showed distinct enzyme activity that converts BBR to D-BBR and high sequence similarity to the bacterial tetrahydrofolate (THF)-dependent aminomethyltransferase of the glycine cleavage system’s T-protein. Because of the high sequence similarity of the demethylase and demethylenases isolated here, we discuss the common origin of these THF-dependent enzymes that degrade plant-specific secondary metabolites in bacterial cells.

## Results

### Isolation and characterization of *brdA*

**Figure 2:**
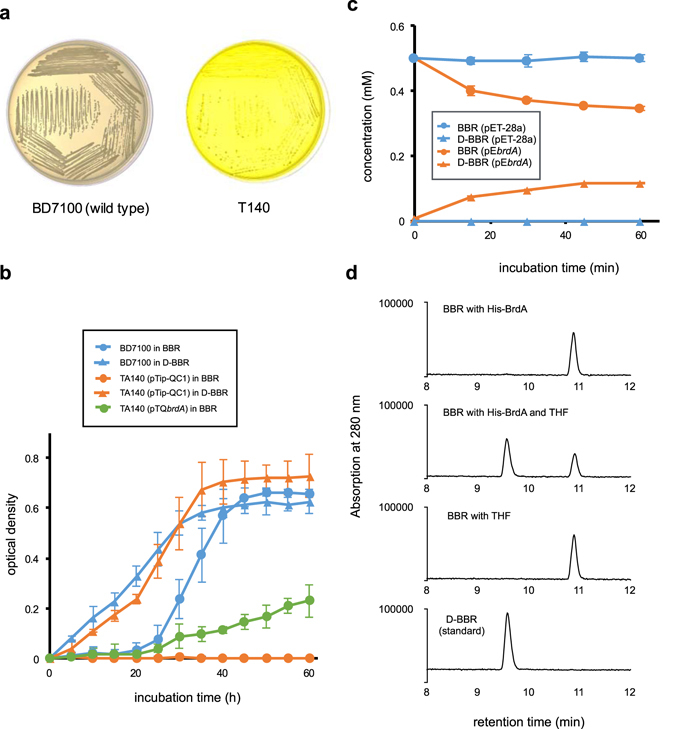
Characterization of BrdA. (**a**) Growth of BD7100 and the transposon mutant TA140 for 5 days on LB medium containing 0.5 mM BBR. Compared with wild-type BD7100 strains, the BBR-derived yellow color in the medium did not disappear with TA140. (**b**) The growth of BD7100 and TA140 in minimal media containing BBR (circles) or D-BBR (triangles). The blue and orange lines indicate growth of BD7100 and TA140 harboring pTip-QC1 (vector control), respectively. The green line indicates growth of TA140 cells harboring pTQ*brdA*. Each value is the average of at least three measurements. The vertical lines indicate the standard deviations from the means. The addition of the *brdA* gene to the TA140 strain restored growth in BBR. (**c**) Crude-extract assay of BBR. The blue and orange lines indicate crude extracts of *E. coli* BL21 (DE3) harboring pET-28a and pE*brdA*, respectively. Circles and triangles indicate concentrations of BBR and D-BBR, respectively. Each value is the average of at least three measurements. The vertical lines indicate the standard deviations from the means. (**d**) HPLC chromatograms (UV 280 nm) of the reaction at 10 min in the purified-enzyme assay. The D-BBR standard is shown at the bottom. The production of D-BBR is observed only in the presence of THF with His-BrdA.

To isolate the genes that are involved in the degradation of BBR, a plasmid for transposon tagging, pTNR-TA, was introduced into BD7100, and some mutant strains that do not exhibit BBR degradation, such as T140, were isolated (Fig. 2a). The transposon insertion site in TA140 was amplified by inverse PCR, sequenced and identified by a BLAST search against the genome sequence of BD7100. The insertion of the transposon was found in the CDS6194 gene, which has been annotated as a vanillate/3-*O*-methylgallate *O*-demethylase in BD7100.

The amino acid sequence of CDS6194 has 44–50% identity with the T-protein of the glycine cleavage system (GcvT), which is expected to function as a THF-dependent aminomethyltransferase in various bacteria (Supplementary Table 1). In addition, the amino acid sequence of CDS6194 has 26.7% identity with the THF-dependent enzymes LigM (vanillate demethylase, accession: BAD61059) and DesA (syringate demethylase, accession: BAC79257), which are reported to function in lignin degradation by *Sphingobium paucimobilis* strain SYK-6^13, 14^. The sequence is also 27% identical to the THF-dependent enzyme SesA (accession: LC101493), a sesamin (a lignan) demethylenase, which was reported in the sesamin-metabolizing bacterium *Sinomonas* sp. no. 22^15^. Reverse transcription PCR (RT-PCR) analysis of the CDS6194 gene in BD7100 (Supplementary Fig. 1) indicated that its transcription only occurs in BD7100 cells that are grown in LB medium containing BBR, which suggests the active involvement of CDS6194 in the degradation of BBR in BD7100.

The function of CDS6194 was further confirmed using resting-cell assays of TA140 and comparing the results to those of BD7100 (Supplementary Fig. 2). When the resting cells were grown in LB medium containing BBR (BBR-induced cells), BBR was almost completely degraded within 1.5 h; the production of D-BBR was observed in BD7100 cells, but TA140 did not degrade BBR or produce D-BBR. Thus, we named the CDS6194 gene ‘*brdA*’ (berberine degrading gene A).

To confirm the function of *brdA*, TA140 cells were complemented with *brdA* by using pTQ*brdA* and the growth were observed in the minimal medium containing BBR or D-BBR (Fig. 2b). Whereas TA140 cells that contained pTip-QC1 (the vector control) did not grow on BBR, TA140 cells containing pTQ*brdA* demonstrated the ability to grow on BBR. However, both BD7100 and TA140 were able to grow on D-BBR, suggesting that BrdA is essential for the conversion of BBR to D-BBR.

To determine the enzymatic activity of BrdA in BD7100, BrdA with a 6xHis-tag at its N-terminus (pE*brdA*) was overexpressed in *E. coli* BL21 (DE3). Crude-extract assays with BrdA degraded 24% of the BBR within 60 min (Fig. 2c), whereas the negative control harboring pET-28a (+) showed no BBR degradation. The His-BrdA that was purified from the crude *E. coli* extract (Fig. 2d) did not convert BBR to D-BBR, but the addition of THF to the purified BrdA activated the conversion of BBR to D-BBR. This result indicates that BrdA is a THF-dependent enzyme and cleaves the methylenedioxy ring of BBR by transferring the methylene residue to THF.

### Isolation of new BBR-utilizing bacteria

Because D-BBR is a common metabolite of BBR that is found in both BD7100 and BD3100^11, 12^, other BBR-utilizing bacteria were isolated to investigate the significance of demethylenation enzymes in BBR degradation in bacteria. Two BBR-degrading strains that could utilize BBR as a sole carbon source, named GBD-1 and CJ1, were isolated. The 16 S rRNA nucleotide sequences of GBD-1 and CJ1 had high identity with Gram-positive bacteria of the genus *Arthrobacter* and Gram-negative bacteria of the genus *Burkholderia*, respectively. Phylogenetic trees of each BBR-degrader are shown in Supplementary Figs 3 and 4.

### *brdA* homologs in BBR-utilizing bacteria

Detection of D-BBR in GBD-1 and CJ1 cultures confirmed that conversion of BBR to D-BBR was the initial step in BBR degradation in both strains (Supplementary Fig. 5), suggesting that GBD-1 and CJ1 each contain a gene that is similar to *brdA*. BrdA homologs were identified by the amino acid sequences based on the draft genome sequences of each strain (Table 1). The amino acid sequences CDS1201, CDS1137 and CDS4435 shared 29–30% identity with the BrdA of BD7100, whereas the amino acid sequences CDS7326, CDS7349 and CDS4430 shared 59–60% identity. The similarity and identity matrices of the amino acid sequence of BrdA are shown in Supplementary Table 2. The alignment of the amino acid sequences is shown in Supplementary Fig. 6. BD3100, GBD-1 and CJ1 each carried two orthologs of *brdA*.

**Table 1:** Characterization of BrdA homologs in BBR-utilizing bacteria.

strain and CDS number	gene name	size (bp)	amino acid residues (aa)	identity* (%)	annotation	activity** (U)	relative activity (%)
*Rhodococcus* sp. BD7100							
6194	*brdA*	1,374	457	100	vanillate/3-*O*-methylgallate *O*-demethylase	10.9 ± 0.32***	100
*Sphingobium* sp. BD3100							
1201	*brdA1*	1,392	463	34	vanillate/3-*O*-methylgallate *O*-demethylase	5.7 ± 0.35	52 ± 1.69
1137	*brdA2*	1,371	456	33	vanillate/3-*O*-methylgallate *O*-demethylase	4.6 ± 0.17	42 ± 2.36
*Arthrobacter* sp. GBD-1							
4430	*brdA*	1,299	432	61	vanillate/3-*O*-methylgallate *O*-demethylase	10.4 ± 0.59	94 ± 5.35
4435	—	1,431	476	33	vanillate/3-*O*-methylgallate *O*-demethylase	0.1 ± 0.03	1 ± 0.31
*Burkholderia* sp. CJ1							
7326	*brdA1*	1,290	429	61	vanillate/3-*O*-methylgallate *O*-demethylase	8.1 ± 0.26	74 ± 0.23
7349	*brdA2*	1,317	438	60	vanillate/3-*O*-methylgallate *O*-demethylase	6.3 ± 0.21	58 ± 0.42

^*^Amino acid sequence identity with BrdA of BD7100.

**One unit of BBR-degrading activity was defined as degradation of 1 µM BBR per minute using 5 µg/ml of purified His-BrdA enzyme.

Each value is the average of triplicate measurements.

*** ±Indicate standard error of triplicate measurements.

RT-PCR analysis (Supplementary Fig. 7) indicated the presence of transcripts of every homolog except for CDS4335 when the organisms were grown in LB medium containing BBR. Thus, CDS1201, CDS1137, CDS4430, CDS7326 and CDS7349 were named as detailed in Table 1, and their enzymatic activities were examined with mutated cells and recombinant proteins.

As BD3100 has two homologous genes, *brdA1* and *brdA2*, compared with the single *brdA* gene in BD7100, we examined the role of each gene in BBR degradation in BD3100 by constructing deletion mutants using homologs recombination. Whereas each mutant, designated BD∆*brdA1* and BD∆*brdA2*, produced D-BBR in a resting-cell assay (Supplementary Fig. 8), the double-deletion mutant of *brdA1* and *brdA2*, designated BD∆*brdA12*, did not produce D-BBR in the assay (Supplementary Fig. 9). These results indicate that both *brdA1* and *brdA2* of BD3100 function in the cleavage of the methylenedioxy ring of BBR.

We constructed six plasmids (pE1201, pE1137, pE4430, pE4435, pE7326 and pE7349) and expressed each of the BrdA proteins in *E. coli* BL21 (DE3). Although most recombinant proteins were insoluble, several soluble proteins were detected and purified by Ni-NTA resin (Supplementary Fig. 10). The relative activities differed among BrdA homologs; BrdA of BD7100 and GBD-1 showed the highest BBR degradation activity (Table 1). These results clearly indicate that each BrdA protein functions as a BBR-demethylenation enzyme and cleaves the methylenedioxy ring of BBR, with the exception of the CDS4335 gene product.

### Phylogenetic analysis of THF-dependent demethylase and demethylenase

**Figure 3:**
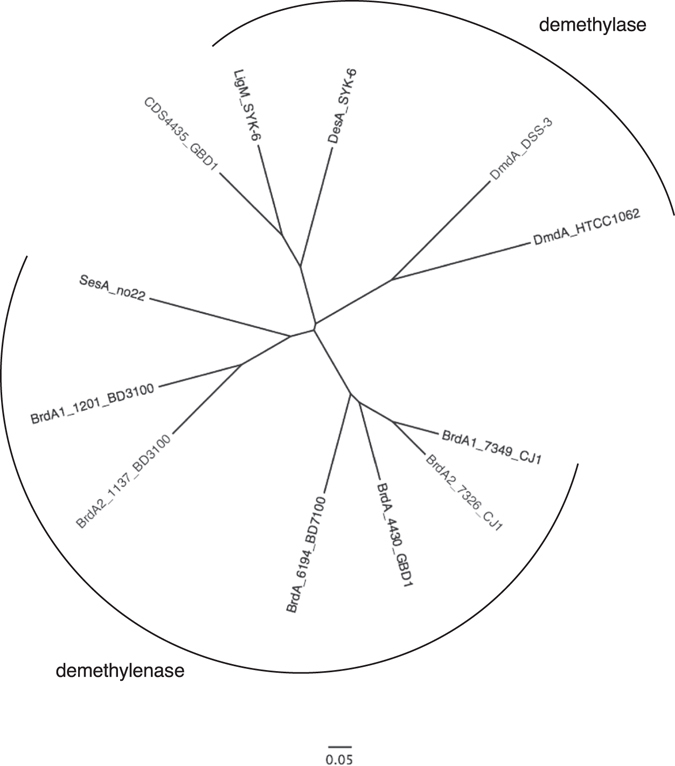
Phylogenetic tree of amino acid sequences in THF-dependent demethylenases and demethylases. The multiple alignments were generated by ClustalW, and phylogenetic trees were constructed using the neighbor-joining method. The amino acid sequences were obtained from NCBI. Enzymes: DmdA_DSS3, DMSP demethylase of *Ruegeria pomeroyi* strain DSS3 (accession: AAV95190); DmdA_HTCC1062, DMSP demethylase of *Candidatus Pelagibacter ubique* strain HTCC1062 (AAZ21068); DesA, syringate demethylase of *S. paucimobilis* SYK-6 (BAC79257); LigM, vanillate demethylase of SYK-6 (BAD61059); and SesA, sesamin demethylenase of *Sinomonas* sp. no. 22 (LC101493). The scale corresponds to a genetic distance of 0.05 substitutions per position.

We isolated four independent BBR-degrading bacteria, *Rhodococcus* sp. BD7100, *Sphingobium* sp. BD3100, *Arthrobacter* sp. GBD-1 and *Burkholderia* sp. CJ1, that belong to different genera. All BBR-degraders contained a *brdA* gene that they used to degrade BBR to D-BBR by demethylenation. Interestingly, BrdA proteins of these BBR-degrading bacteria are similar to the THF-dependent GcvT protein of various bacteria. Among the THF-dependent GcvT homologous enzymes, only four enzymes (SesA from *Sinomonas* sp. no. 22, DesA and LigM from *Sphingobium paucimobilis* SYK-6 and DmdA from *Candidatus Pelagibacter ubique* HTCC1062) have been characterized. DesA, LigM and DmdA catalyze the demethylation of syringic acid, vanillic acid and dimethylsulfoniopropionate (DMSP), respectively^13, 14, 16^. SesA is a demethylenation enzyme, but its substrates are lignans such as sesamin, (−)-asarinin, sesaminol and sesamolin^15^ instead of alkaloids. Phylogenetic tree analysis of these THF-dependent demethylases and demethylenases showed that SesA and BrdA formed a clan of demethylenases, whereas DesA, LigM and DmdA formed another clan of demethylases that includes CDS4435 (Fig. 3). This result indicates the common origin of demethylenases and demethylases. This result also indicates that THF-dependent enzymes have key roles in the degradation of natural products with *O*-methylation or a methylenedioxy ring, such as lignin and lignan. BrdA is likely to be the predominant enzyme in the degradation of BBR.

We compared the amino acid sequences of BrdA homologs and SesA due to their reactive similarity as demethylenases. All the proton donor residues of SesA (R81, H82, R100, R179, and H225), which are expected to be used for demethylenation of the methylenedioxy ring of sesamin, were conserved in all the BrdA proteins except for R81of SesA (Supplementary Fig. 11). Among the residues that were predicted in SesA to make hydrogen bonds with THF (D95, E189, and Y221), E189 of SesA was conserved in all the BrdA proteins. D95 of SesA was conserved in BrdA of BD7100, BrdA of GBD-1, and BrdA2 of BD3100. Y221 was conserved in BrdA1 and BrdA2 of BD3100. None of the BrdA proteins cleaved the methylenedioxy ring of sesamin (data not shown).

## Discussion

In this study, we demonstrated that four species of BBR-degrading bacteria (two Gram-negative and two Gram-positive bacteria) harbor enzymes that cleave the methylenedioxy ring of BBR; these enzymes are similar to GcvT, the THF-dependent demethylenase that is involved in glycine metabolism. Cleavage at the methylenedioxy ring of sesamin in a Gram-positive bacterium, *Sinomonas* sp. no. 22, also occurred. However, demethylenation enzymes that are involved in the cleavage of the methylenedioxy ring of BBR are different in mammals^7–10^, plants^17^ and bacteria. In eukaryotes, enzymatic reactions for BBR metabolism are oxidative reactions that use oxygen and the iron at the active site of their enzymes; both oxygen and iron are required for electron transfer with P450 reductase. Cytochrome P450 is responsible for the demethylenation of a methylenedioxy ring^18–24^. Conjugating BBR is the first step of the excretion process. By contrast, bacteria that have evolved to utilize BBR might have developed GcvT-like enzymes for demethylation and demethylenation without oxidation. We also found that all our isolated BBR-degraders performed demethylenation as the first step of BBR degradation. This sequential process suggests that demethylenation might be an important strategy for all BBR-utilizing bacteria before they proceed to the next step of the BBR degradation process. Our finding of the involvement of *brdA* orthologs in all BBR-degrading bacteria led to the discovery that the initial key reaction for BBR degradation is shared among all bacteria that are capable of BBR degradation. The bacterial demethylenation of methylenedioxy rings is derived from the C1 metabolism of the glycine cleavage system, which is responsible for fundamental metabolism that is common to both prokaryotes and eukaryotes.

## Methods

### Bacterial strains, plasmids and primers

The bacterial strains and plasmids used in this study are described in Supplementary Table 3. pK18*mobsacB* ^25^ was obtained from the National BioResource Project (NIG, Japan): *E. coli*. The rhodococcal transposon vector pTNR-TA^26^ and the expression vector pTip-QC1^27^ were kind gifts from Dr. Tamura. The primers used in this study are listed in Supplementary Table 4.

### Chemicals

Berberine sulfate was purchased from Tokyo Kasei Kogyo Co. (Tokyo, Japan). Demethyleneberberine was chemically synthesized from BBR as described previously^11, 12^.

### Media and culture conditions

Bacteria were grown in LB medium (10 g Bacto-tryptone, 5 g Bacto-yeast extract, and 5 g NaCl, in 1 liter), *Pseudomonas* medium^28^ and WM medium [1.7 g KH_2_PO_4_, 12.4 g Na_2_HPO_4_ · 12H_2_O, 1.0 g (NH_4_)_2_SO_4_, 2.0 mg CaCO_3_, 1.4 mg ZnSO_4_ · 7H_2_O, 0.25 mg CuSO_4_ · 5H_2_O, 0.28 mg, CoSO_4_ · 7H_2_O, 0.06 mg H_3_BO_4_, 100 mg MgSO_4_ · 7H_2_O, 0.95 mg FeSO_4_ · 7H_2_O and 10.7 mg MgO in 1 liter]. All cultures were incubated at 30 °C, and liquid cultures were aerobically incubated with shaking at 180 rpm. Growth was monitored using an OD monitor (TAITECH, Tokyo, Japan) to measure the absorption at 600 nm.

### Isolation of transposon mutants

One microgram of pTNR-TA was electroporated (voltage: 1200 V, capacitance: 25 µF, resistance: 800 Ω) in 0.1 ml of BD7100 cells in a chilled cuvette (1 mm gap) with a Gene Pulser II (Bio-Rad). One ml of SOC medium was then added to the cuvette immediately. The cell suspension was transferred to a sterile tube and heat shocked at 42 °C for 2 min. After incubation at 30 °C for 6 h, cells were spread onto LB agar plates containing 2 µg/ml thiostrepton. Each transformant was transferred to 0.2 ml of LB medium that contained 2 µg/ml thiostrepton and 0.5 mM BBR in a microtiter plate and incubated at 30 °C for five days. The transformants that did not bleach the yellow color derived from BBR were streaked onto 0.1x LB agar plates containing 2 µg/ml thiostrepton and 0.5 mM BBR.

### Determination of transposon insertion sites

Total DNA from transposon mutants was extracted from each bacterial pellet with a Genomic DNA Purification Kit (Promega KK, Tokyo, Japan), according to the manufacturer’s protocol. The total DNA was digested by *Hin*dIII (NEB, Tokyo, Japan), followed by heat inactivation of the restriction enzyme and self-ligation by Ligation high (TOYOBO, Osaka, Japan). The ligated DNA solution was used as the inverse PCR template. The inverse PCR was performed using KOD plus neo DNA polymerase (TOYOBO), using primers TNR-TAinvFw and TNR-TAinvRv (Supplementary Table 4), with the following program: initial denaturation at 96 °C for 120 sec, 40 cycles of amplification (98 °C for 20 sec, 55 °C for 20 sec and 68 °C for 45 sec) and extension at 68 °C for 5 min. Amplified products were purified by the Wizard SV Gel and PCR Clean-Up System (Promega KK), according to the manufacturer’s protocol. The purified DNA fragments and TNR-TAinvRv primers were used for sequencing. The sequencing reactions were performed using the Big Dye Terminator Cycle Sequencing system (Applied Biosystems, CA, USA) on an automated ABI 3100 sequencer (Applied Biosystems). The nucleotide sequence was submitted to a BLAST search of the genome sequence of BD7100 in the RAST server.

### Resting-cell assay

Bacterial cells were grown at 30 °C with shaking for 16 h in 100 ml of LB medium containing 0.5 mM BBR to make resting cells, which include enzymes induced by BBR. The cells were collected by centrifugation (2,610 × *g*, 15 min, room temperature), washed twice with 50 mM sodium phosphate buffer (pH 7.0) and then suspended in 20 ml of 50 mM sodium phosphate buffer to yield a turbidity of 10 at 600 nm. Substrate was added to a final concentration of 0.5 mM, and the mixture of resting-cell suspension and substrate in the sodium phosphate buffer was incubated at 30 °C with shaking after adding the appropriate antibiotics. Aliquots of the reaction (0.4 ml/time point) were periodically removed, and the reaction was stopped (cells lysed, reactions quenched) by the addition of an equal volume of stop solution (MeOH containing 0.05 N HCl). After centrifugation at 18,800 × *g* at 4 °C for 10 min, the supernatants were analyzed by high-performance liquid chromatography (HPLC). HPLC conditions were described in a previous study^11, 12^.

### Reverse transcription-polymerase chain reaction (RT-PCR)

Bacterial cells were grown in 10 ml of LB or LB containing 0.5 mM BBR at 30 °C for 18 h with shaking. The cells were collected by centrifugation and washed three times with TE buffer. Total RNA was isolated using ISOGEN (Nippon gene, Tokyo, Japan), according to the manufacturer’s protocol. RT-PCR was performed using the ReverTra Ace qPCR RT Master Mix with gDNA Remover (TOYOBO Co., Ltd., Osaka, Japan), as described in the manufacturer’s protocol. One microgram of total RNA was reverse-transcribed with random primers, and 32 cycles of PCR were performed (98 °C for 20 sec, 55 °C for 20 sec and 68 °C for 45 sec) with the primer sets listed in Supplementary Table 4.

### Complementation of *brdA* in TA140

To produce pTQ*brdA*, a *Nco*I-*Hin*dIII fragment PCR product was generated with primers CDS6194NcoFw and CDS6194HinRv (Supplementary Table 4) and cloned into the *Nco*I-*Hin*dIII site of pTip-QC1. pTip-QC1 and pTQ*brdA* were introduced into TA140 cells by electroporation (voltage: 1200 V, capacitance: 25 µF, resistance: 800 Ω, cuvette: 1 mm). Each transformant was precultured in LB medium containing 2 µg/ml thiostrepton and 30 µg/ml chloramphenicol. One milliliter of preculture was added to 100 ml of WM medium containing 5 µg/ml thiostrepton, 50 µg/ml chloramphenicol and 0.5 mM BBR or D-BBR and incubated at 30 °C with shaking. Growth was monitored using an OD monitor (TAITECH, Tokyo, Japan), and absorption was measured at 600 nm.

### Expression of BrdA homologs with an N-terminal His-tag in *E. coli*

The cloning methods for construction of pET system plasmids are described in the Supplementary Methods. Each plasmid was introduced into *E. coli* BL21 (DE3) by electroporation (voltage: 1800 V, capacitance: 25 µF, resistance: 200 Ω, cuvette: 1 mm), and the transformants were incubated in 100 ml of LB medium containing 25 µg/ml kanamycin at 15 °C, with shaking at 150 rpm. Isopropyl-β-D-thiogalactopyranoside (IPTG) (0.1 mM) was added to the culture when the OD_600_ of the culture reached 0.5. After 17 h of incubation, cells were collected by centrifugation and washed in PBS buffer three times. Washed cells were suspended in PBS and ruptured by an ultrasonic cell disruptor. The solution was centrifuged at 18,800 × *g* for 10 min to separate the cell body and crude enzyme extract, and the supernatant was obtained as the crude extract, which contained His-BrdA. As a negative control, the crude extract without His-BrdA was prepared from cells of *E. coli* BL21 (DE3) harboring pET-28a ( + ) under the same conditions.

### Crude-extract assay

The concentration of each protein was measured using a Qubit protein assay kit and Qubit 2.0 (Invitrogen, Carlsbad, CA, USA). The crude extract (1.2 mg of protein) was added to 0.5 mM substrate in 50 mM Tris-HCl buffer (pH 7.0) and incubated at 30 °C. Fifty microliters of reaction solution was collected at 15-min intervals, and the reaction was stopped by adding an equal volume of stop solution. The amounts of BBR and D-BBR in the mixture were quantified by HPLC.

### Purification of His-BrdA

His-BrdA proteins in the crude extract were purified using HisPur^TM^ Ni-NTA Resin (Thermo Fisher Scientific, Yokohama, Japan), according to the manufacturer’s protocol, and the sample in the elution buffer was exchanged into 50 mM Tris-HCl buffer using a Microcon centrifugal filter device (Millipore, Bedford, MA, USA). The concentration of purified proteins was measured using a Qubit protein assay kit and Qubit 2.0 (Invitrogen, Carlsbad, CA, USA). The expression and purity of each His-BrdA protein were assessed by SDS-PAGE.

### Purified-enzyme assay

The assay was performed in 500 µl of 50 mM Tris-HCl buffer (pH 7.0) containing 10 µg of His-BrdA, 0.5 mM BBR and 2 mM THF. THF was dissolved in a solution containing 0.5 mM Tris-HCl buffer (pH 9.0), 1% (v/v) 2-mercaptoethanol and 2% (w/v) ascorbate. The reaction was stopped by adding an equal volume of stop solution. The concentrations of BBR and D-BBR were analyzed by HPLC.

### Isolation of BBR-utilizing bacteria

A piece of root from *Coptis japonica* (15 mm) with soil was added directly to 3 ml of WM medium containing 0.5 mM BBR and incubated at 30 °C for 6 days in a shaker. An aliquot of the resulting culture was diluted 1:100 into 3 ml of fresh WM medium containing 0.5 mM BBR, and the dilution was incubated at 30 °C for 6 days on a rotary shaker. This 1:100 dilution and outgrowth was repeated twice more (for a total of three times). An aliquot of the resulting culture was spread directly onto a plate of WM medium agar containing 0.5 mM BBR.

### Identification of BBR-utilizing bacteria

Total DNA of BD3100 and BD7100 was extracted using a Genomic DNA Purification Kit (Promega KK, Tokyo, Japan), according to the manufacturer’s protocol. The 16 S rDNA was amplified using the 27 F and 1525 R primer pair (Supplementary Table 4). PCR was performed using KOD plus neo DNA polymerase (TOYOBO, Osaka, Japan) with the following program: initial denaturation at 96 °C for 120 sec, 34 cycles of amplification (94 °C for 20 sec, 55 °C for 20 sec and 68 °C for 45 sec) and extension at 68 °C for 5 min. Amplified products were purified using the Wizard SV Gel and PCR Clean-Up System (Promega KK). The purified DNA fragments were used a template for sequencing reactions, which were performed with 27 F or 1525 R primers using the Big Dye Terminator Cycle Sequencing system (Applied Biosystems, CA, USA) on an automated ABI 3100 sequencer (Applied Biosystems). The 16 S rDNA gene sequences of the isolated bacteria were submitted to the classifier program in the Ribosomal Database Project, release 11.3, which is maintained by the Center for Microbial Ecology at Michigan State University (http://rdp.cme.msu.edu/)^23^.

### Construction of deletion mutants of BD3100 by homologous recombination

The deletion mutants of target genes were constructed by double crossover using pK18*mobsacB*. Details of the construction of pK18∆1201 and pK18∆1137 are described in the Supplementary Methods. The plasmids pK18∆1201 and pK18∆1137, derived from pK18*mobSacB*, were electroporated into BD3100. The transformants were selected on LB plates containing 50 µg/ml of kanamycin. To make a double-crossover mutant, the sucrose-sensitive transformants were selected and incubated in LB for 12 h. The sucrose-resistant mutants were selected on LB plates containing 10% sucrose. The mutants that showed the sucrose-resistant and kanamycin-sensitive phenotype were candidates for deletion mutants. The gene deletion was confirmed by Southern hybridization (Supplementary Fig. 12). To make 1202 and 1138 probes, PCR was carried out using the 1202 F and 1202 R primer set and the 1138 F and 1138 R primer set, respectively (Supplementary Table 4). The labeling of PCR products and Southern hybridization were performed using a DIG-High Prime DNA Labeling and Detection Starter Kit (Roche), according to the manufacturer’s protocol.

### Data Availability

The nucleotide sequences reported in this study have been deposited in the International Nucleotide Sequence Database Collaboration under Accession No. LC214531 for 16 S rRNA of GBD-1, LC214532 for 16 S rRNA of CJ1, LC214741 for *brdA* of BD7100, LC214742 and LC214743 for *brdA1* and *brdA2* of BD3100, respectively, LC214744 and LC214745 for *brdA* and CDS4435 of GBD-1 and LC214746 and LC214747 for *brdA1* and *brdA2* of CJ1.

## Electronic supplementary material

Supplementary Information
