# Supplementary material for: Common origin of methylenedioxy ring degradation and demethylation in bacteria
Source: Sci Rep. 2017 Aug 7;7:7422. doi: 10.1038/s41598-017-07370-x (PMC5547118; doi:10.1038/s41598-017-07370-x)
Supplement: Supplementary file 1 — Supplementary Information [file 41598_2017_7370_MOESM1_ESM.pdf]

Common origin of methylenedioxy ring degradation and demethylation in bacteria

Hisashi Takeda, Kazuki Ishikawa, Hinaka Yoshida, Daisuke Kasai, Daigo Wakana, Masao Fukuda, Fumihiko Sato, and Tomoo Hosoe

Table of contents

Supplementary Methods

Plasmid construction

Supplementary Figures

Supplementary Figure 1 to 12

Supplementary Tables

Supplementary Table 1 to 4

Supplementary Methods

Plasmid construction

[pE*brdA*] The 1,317 bp DNA fragment carrying *brdA* of BD7100 was prepared by PCR with primers ETNde6194Fw and ETHin6194Rv (Supplementary Table 4), adding *Nde*I and *Hin*dIII sites, respectively. The PCR product was cloned into the SmaI site of pUC19 to generate pUbrdAE. pUbrdAE was digested with *Nde*I and *Hin*dIII, and the fragment carrying *brdA* was cloned into the *Nde*I and *Hin*dIII sites of pET-28a to generate pE*brdA*.

[pE1201] The 1,412 bp DNA fragment carrying *brdA1* of BD3100 was produced by PCR with primers ETBam1201Fw and ETHin1201Rv (Supplementary Table 4), adding *Bam*HI and *Hin*dIII sites, respectively. The PCR product was cloned into the *Sma*I site of pUC19 to generate pU1201. pU1201 was digested with *Bam*HI and *Hin*dIII, and the fragment carrying *brdA1* was cloned into the *Bam*HI and *Hin*dIII sites of pET-28a to generate pE1201.

[pE1137] The 1,383 bp DNA fragment carrying *brdA2* of BD3100 was produced by PCR with primers ETBam1137Fw and ETHin1137Rv (Supplementary Table 4), adding *Bam*HI and *Hin*dIII sites, respectively. The PCR product was cloned into the *Sma*I site of pUC19 to generate pU1137. pU1137 was digested with *Bam*HI and *Hin*dIII, and the fragment carrying *brdA2* was cloned into the *Bam*HI and *Hin*dIII sites of pET-28a to generate pE1137.

[pE4430] The 1,301 bp DNA fragment carrying *brdA* of GBD-1 was produced by PCR with primers ETEco4430Fw and ETHin4430Rv (Supplementary Table 4). The PCR product was cloned into the *Sma*I site of pUC19 to generate pU4430. pU4430 was digested with *Eco*RI and *Hin*dIII, and the fragment carrying *brdA* was cloned into the *Eco*RI and *Hin*dIII sites of pET-28a to generate pE4430.

[pE4435] The 1,419 bp DNA fragment carrying CDS4435 of GBD-1 was produced by PCR with primers ETNde4435Fw and ETHin4435Rv (Supplementary Table 4), in which ETNde4435Fw added an *Nde*I site. The PCR product was cloned into the *Sma*I site of pUC19 to generate pU4435. pU4435 was digested with *Nde*I and *Hin*dIII, and the fragment carrying CDS4435 was cloned into the *Nde*I and *Hin*dIII sites of pET-28a to generate pE4435.

[pE7326] The 1,320 bp DNA fragment carrying *brdA1* of CJ1 was produced by PCR with primers ETNde7326Fw and ETHin7326Rv (Supplementary Table 4), in which ETNde7326Fw added an *Nde*I site. The PCR product was cloned into the *Sma*I site of pUC19 to generate pU7326. pU7326 was digested with *Nde*I and *Hin*dIII, and the fragment carrying *brdA1* of CJ1 was cloned into *Nde*I and *Hin*dIII sites of pET-28a to generate pE7326.

[pE7349] The 1,293 bp DNA fragment carrying *brdA2* of CJ1 was produced by PCR with primers ETNde7349Fw and ETHin7349Rv (Supplementary Table 4), in which ETNde7349Fw added an *Nde*I site. The PCR product was cloned into the *Sma*I site of pUC19 to generate pU7349. pU7349 was digested with *Nde*I and *Hin*dIII, and the fragment carrying *brdA2* of CJ1 was cloned into the *Nde*I and *Hin*dIII sites of pET-28a to generate pE7349. Please substitute either "Plasmid construction" or "Plasmid construct" here.

[pK18Δ1201] The 869 bp PCR fragment carrying the 5'-upstream region of *brdA1* of BD3100 was amplified with primer set 1201UPFw and 1201UPRv (Supplementary Table S4), which provided additional *Eco*RI and *Xba*I sites, respectively. The PCR fragment was cloned into the *Sma*I site of pUC19 to produce pU1201UP. The 807 bp PCR fragment carrying the 3'-downstream region of *brdA1* was amplified with primer set 1201DOWNFw and 1201DOWNRv (Supplementary Table S4), which provided additional *Xba*I and *Hin*dIII sites, respectively. The PCR fragment was cloned into the *Sma*I site of pUC19 to produce pU1201DOWN. The *Eco*RI-*Xba*I fragment of pU1201UP was cloned into the *Eco*RI-*Xba*I site of pK18*mobsacB* to produce pK18-1201UP. The *Xba*I-*Hin*dIII fragment of pU1201DOWN was cloned into the *Xba*I-*Hin*dIII site of pK18-1201UP to produce pK18Δ1201.

[pK18Δ1137] The 1,127 bp PCR fragment carrying the 5'-upstream region of *brdA2* of BD3100 was amplified with primer set 1137UPFw and 1137UPRv (Supplementary Table S4), which provided additional *Eco*RI and *Xba*I sites, respectively. The PCR fragment was cloned into the *Sma*I site of pUC19 to produce pU1137UP. The 943 bp PCR fragment carrying the 3'-downstream region of *brdA2* was amplified with primer set 1137DOWNFw and 1137DOWNRv (Supplementary Table S4), which provided additional *Xba*I and *Hin*dIII sites, respectively. The PCR fragment was cloned into the *Sma*I site of pUC19 to produce pU1137DOWN. The *Eco*RI-*Xba*I fragment of pU1137UP was cloned into the *Eco*RI-*Xba*I site of pK18*mobsacB* to produce pK18-1137UP. The *Xba*I-*Hin*dIII fragment of pU1137DOWN was cloned into the *Xba*I-*Hin*dIII site of pK18-1137UP to produce pK18Δ1137.

Supplementary Figures

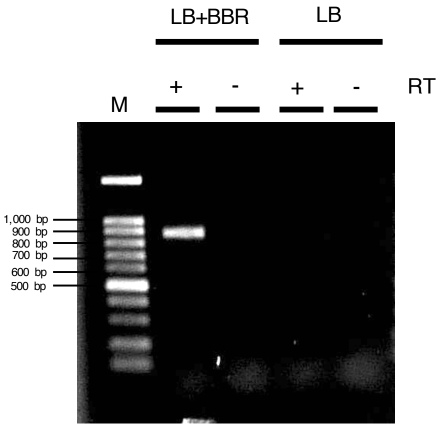

Supplementary Figure 1. Transcription of *brdA* in BD7100.
Transcription of *brdA* in BD7100 was analysed by RT-PCR. Total RNA from *Rhodococcus* sp. BD7100 cells grown on LB or LB containing 0.5 mM BBR was reverse-transcribed. RNA samples were concurrently analysed in PCR mixtures with (+) and without (-) reverse transcriptase (RT) to verify the absence of total DNA. Primer sets (RT6194Fw and RT6194Rv) indicated in Supplementaly Table 4 were designed to amplify cDNAs in the internal region of *brdA*. The lane of the DNA ladder marker is indicated by M. RT-PCR products were detected and corresponded to the predicted size of 884 bp. Since RT-PCR products were only detected in the cells grown with BBR, transcription of *brdA* is induced by BBR in BD7100.

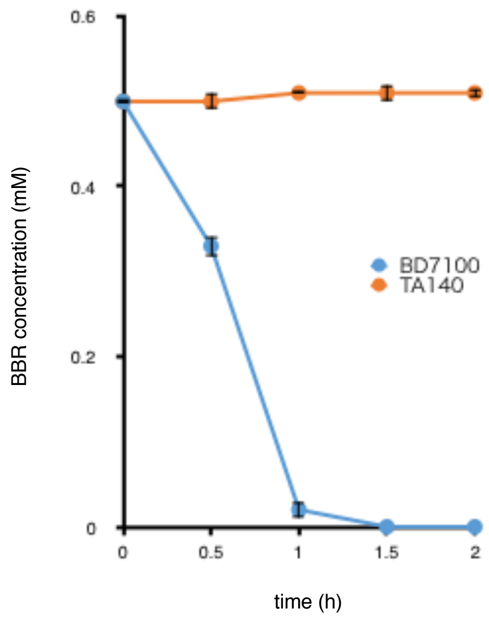

Supplementary Figure 2. BBR degradation in the transposon mutant TA140. Resting-cell assays of BBR were carried out using BD7100 and TA140. The blue and orange lines show BD7100 and TA140, respectively. Each value is the average of at least three measurements. The vertical lines indicate the standard deviations from the means. TA140 lost the ability to degrade BBR.

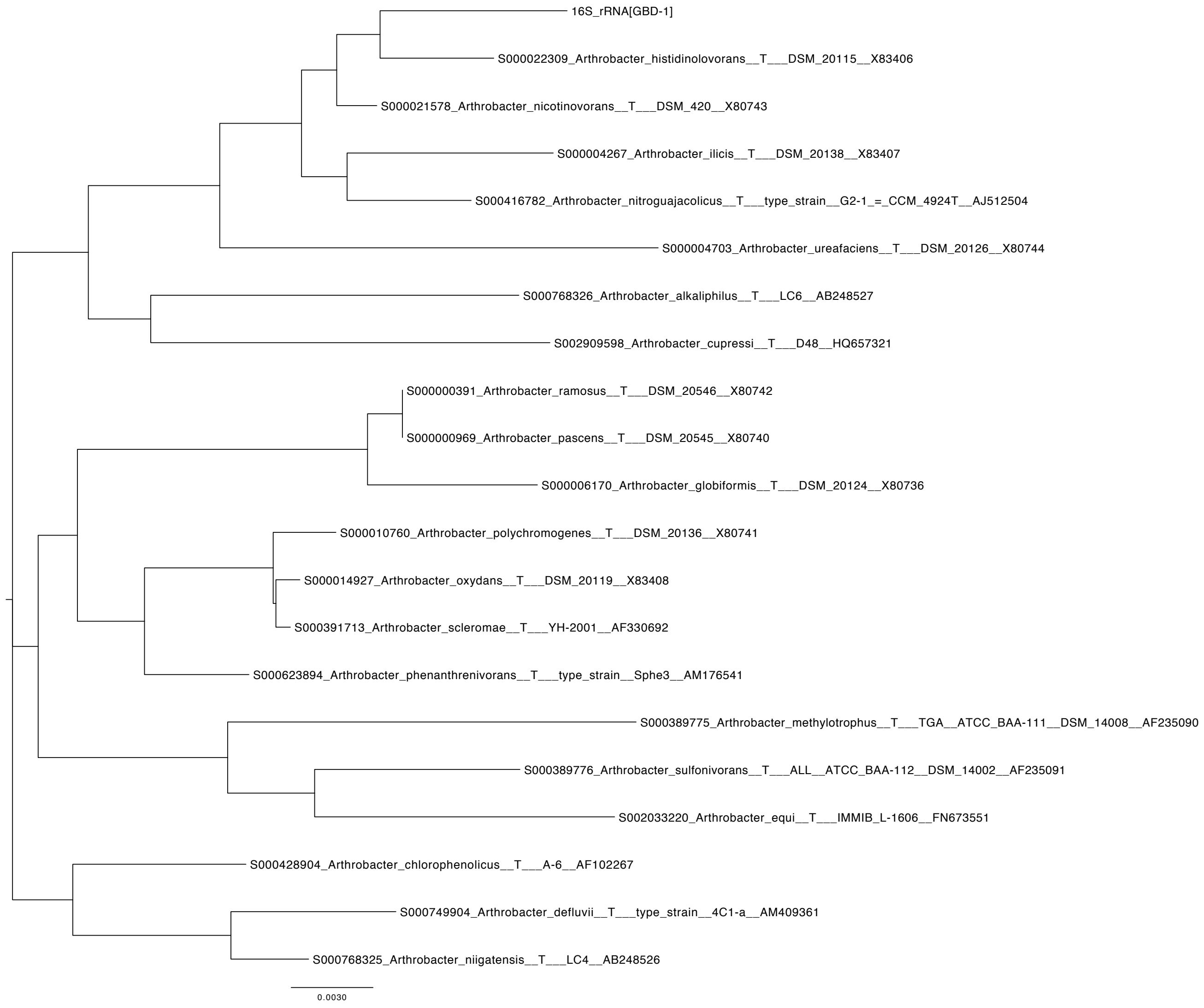

Supplementary Figure 3. **Phylogenetic tree of GBD-1 with strains of the genus *Arthrobacter*.**
The 16S rRNA sequence of GBD-1 was aligned by the Sequence Match application on the RDP website (http://rdp.cme.msu.edu/). Data Set Options were selected as follows: strain, Type; source, Isolates; size, >1200; quality, good; KNN matches, 20. Phylogenetic trees were constructed using the neighbour-joining method with the Kimura method.

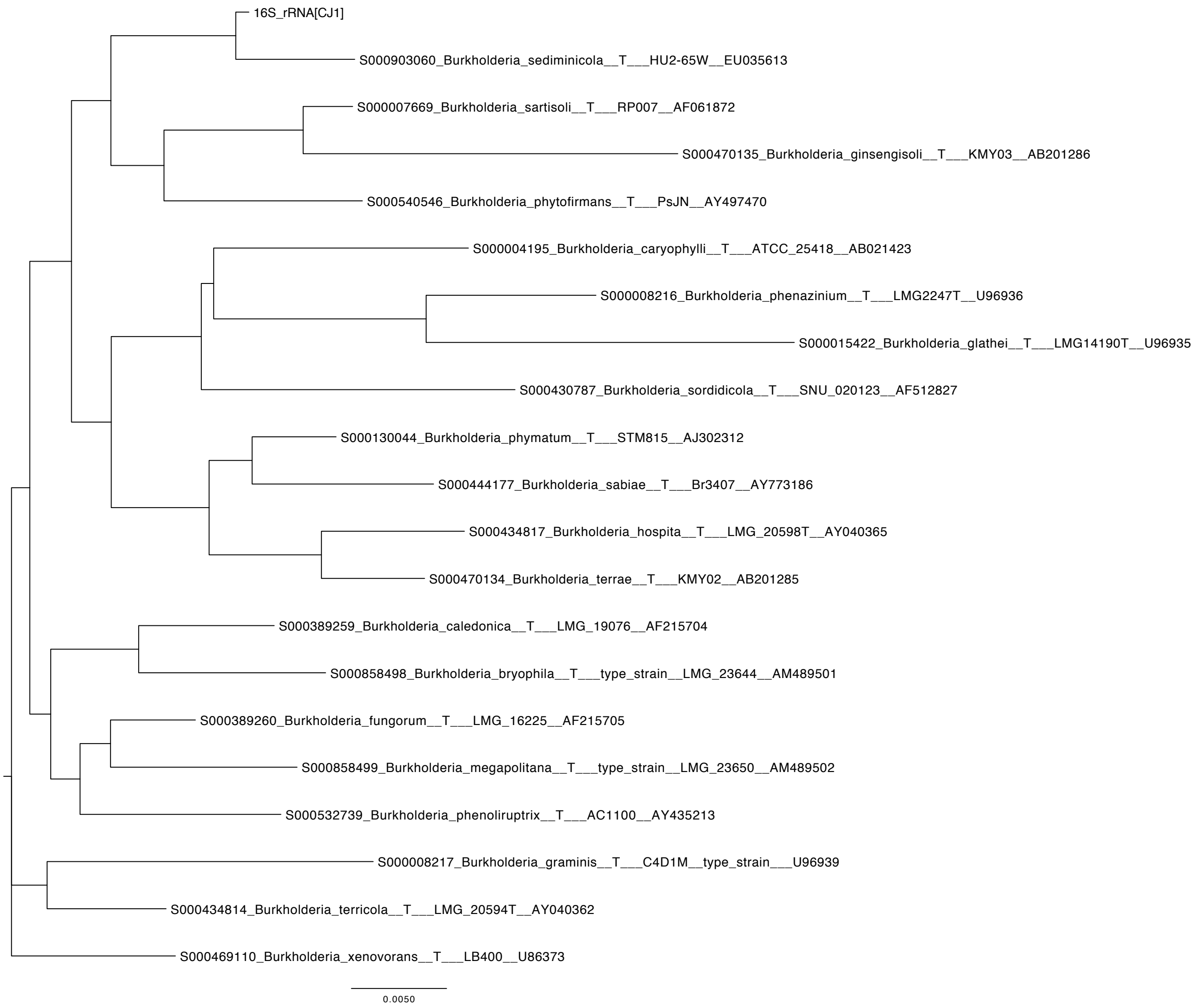

Supplementary Figure 4. Phylogenetic tree of CJ1 with strains of the genus *Burkholderia*.
The 16S rRNA sequence of CJ1 was aligned by the Sequence Match application on the RDP website (http://rdp.cme.msu.edu/). Data Set Options were selected as follows: strain, Type; source, Isolates; size, >1200; quality, good; KNN matches, 20. Phylogenetic trees were constructed using the neighbour-joining method with the Kimura method.

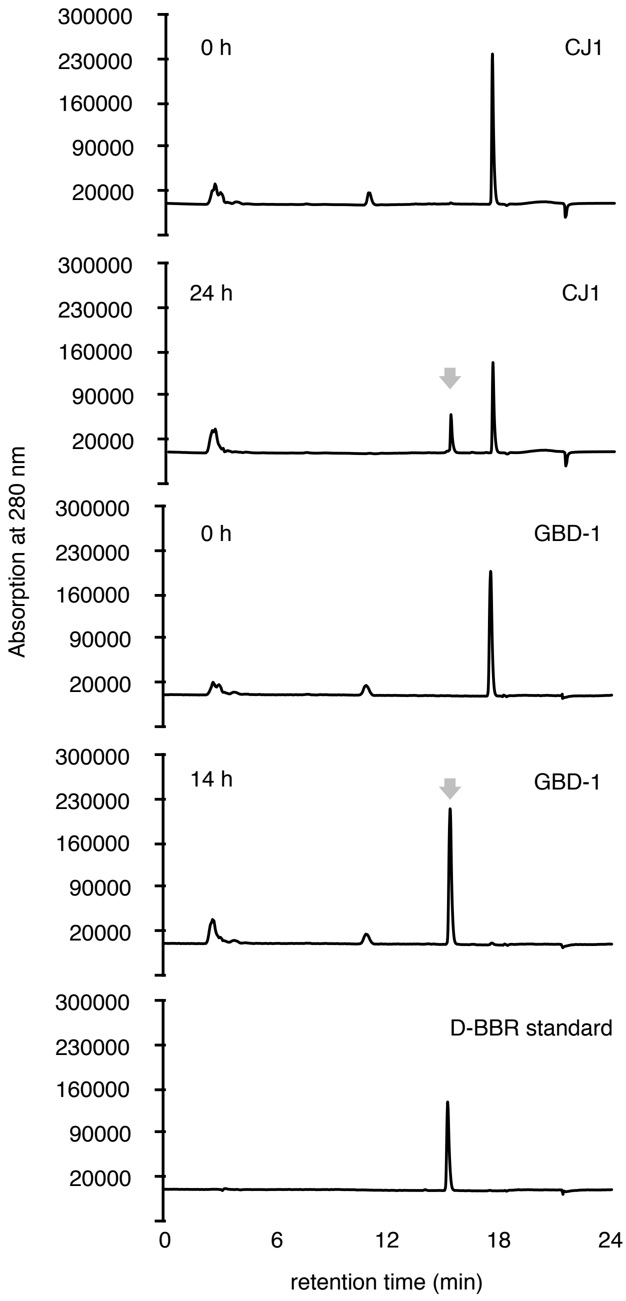

Supplementary Figure 5. Detection of BBR metabolites in GBD-1 and CJ1.
HPLC chromatograms, monitored at 280 nm, of culture medium containing BBR at the initial time and after incubation of GBD-1 and CJ1. GBD-1 and CJ1 were grown in LB medium containing 0.5 mM BBR. The sampling times of the cultures are indicated in the chromatograms. The peaks at 15.3 min retention time, indicated by grey arrows, correspond to the retention time of the standard D-BBR. The detection of D-BBR in all BBR-degraders suggests that demethylenation of BBR is an important step of BBR degradation.

```
BrdA[BD7100]         1 -----------MPSFRDSLVAQLRHPIQFHAGDMWGPPQYTNWMDEERSWKDSCYLGDWTFLP-TIRYTGPDVLKLFADCSVNTMNNFKIGQSKHIIHTNRDGKVIEDGL  98
BrdA[GBD-1]          1 ---------MTQTPSPQFSVSALRNPIREHHGAVWGPPQYTNWIEESLSWKETCYLGDWTFLP-SMRYTGPDVLRLFSDVSVNTMNNFAIGQSKHIIQCDENGKIIDDAV 100
CDS4435[GBD-1]       1 MNVNKPKSLQDVIDSNANIVDVLRNAQNPAYIVPVVPSEFTNWRSEQRAWQETAVLFDQTHHMDNLILRGPDAIKLISDTAVNSVANFDVNKAKQYVATTDTGHVVGDGI 110
BrdA1[CJ1]           1 --------MENSHQKLDFSVKSLRRPLKSHFTDSWGPPQFTNWVDESMSWKETCYLGDWTFLD-ALKYTGPDVLKLFADTSVNTMQNFEIGQSKHVIHCNEDGKIIEEGI 101
BrdA2[CJ1]           1 --------MKTENSTPNLSMASFRRPIRPHFGDAWGPPQFTNWIDESLSWKETCYLGDWTFLP-ALKYTGPDVLKLFADTSVNTMENFAIGQSKHVIHCNEDGKIIEEGI 101
BrdA1[BD3100]        1 ------MTDPTLLPHSPYLPYDQSLLNYNMTYGHLDPMEFGGWKRETLSWKEGCYLHAGLNPPSPYRISGPDAIRLFSDACVNSFAKFSIGGSKHAIMCNAEGNIMAHGM 104
BrdA2[BD3100]        1 -----------MIPHSPYLPFDPDVSDYNVAFTHLSPWEFGGWKRESLSWKKGCYLHAGLNPPSPYRLSGPDALALLRDACINGFSTFSIGCSKHAVMCNAQGNVMADGM  99
                                                          * .. .*  *  .*.  . *           *** . *  * ..*   * .. .*. . .  * ..   .

BrdA[BD7100]        99 LTRTADEELICYSSY----WADYIRRNGNYRVD-------------MEPIEQVKFHLQGPNALFVLETALGRDFRDLKFMRNEDVTIA-----GVPTRVLRQGMSGEIG 185
BrdA[GBD-1]        101 LSRTGEQEVISFSTF----WADYVRRQGDYDVE-------------AEYVGLSKFHLQGPTSLFVLESAAQASVRDLKFMRSMKVSIA-----DVEVTVLRQGMSGEVG 187
CDS4435[GBD-1]     111 LFREAEEEFLYVGRSQAANWLRFHGESGGYNVDIEIDRRSPTNPMGHAVPRKYWRLQIQGPNAWEIIEKVNGGPVEQLKFFNMSHINVA-----GERVRTLRHGMSGVPG 215
BrdA1[CJ1]         102 LSRFGENEYVAFSMY----WADHVRRQGNYDVNP------------PELLPLTKFHLQGPNALFVLEKVANESLRDLKFMRFRKIQIA-----GHEVIALRQGMSGEIG 189
BrdA2[CJ1]         102 LSRFSENEYVAFSMY----WADYVRRQGNYDVEP------------AELLPLTKHHLQGPNALFVLEKVANESLRDLKFMRFRKIRIA-----GHEVLALRQGMSGEIG 189
BrdA1[BD3100]      105 LLRTGEEEFQSFFLSP---YINYLVESGKYDVRGE-----------DMTGKVFMFQVAGPRSLEVIEAATQEDLHDIKFLRHRPSQIVGADGRKIQVRIVRIGMAGTLA 199
BrdA2[BD3100]      100 VLRTGEEDFTCFFLNP---YIDYLAASGRYDVRGE-----------DLSGKVFLFQVAGPRSLEVMEAATGENLRDIEFLWHRASTIR-ADGRDVPVRVFRLGVARTLA 193
                       . *  . .                  * * *                       .. ** .  ..*        . *       .            * *..

BrdA[BD7100]       186 FELQADKAQGQILRETILEAGTKYGIHEMGGRVAMLNHLQAAYPTVMTDYLPAMYDDDGAGYLEEYMGEADGYFARYYGAVAGSFESDDVSGWYRSPVELGWGGRINFDH 295
BrdA[GBD-1]        188 FELQFPIEKADVVRAALLEAGSDHGMKQMGGRVAMLNHLEAYYPTQGLDYMPAVFDGRHPDFLHELVENGGGWMDIYYR-VAGSFESDDIRDWYRSPVEFGWGNRINFDH 296
CDS4435[GBD-1]     216 LELWGAYESYAKVRDAIFDAGQELGLLAAGGRTYPTNTLESGWIP---DPLPAIYTG---ENLRPYREWLGADSAEAKNAIAGSYVSNNIEDYYLTPWELGYGSFVKFDH 319
BrdA1[CJ1]         190 FELQGPLEHREEIWNAIFEAGQEFGIRQMGGRVAMINHLEANYPTNALDYLPAIFDENNSAYLGEMFTNYKELFDYYFR-VAGSYDSSSVADWYRSPVELGWGNRIKFDH 298
BrdA2[CJ1]         190 FELQGPLEHREEIWNTILEAGREFGIRQMGGRVAMINHLEANYPTICLDYMPAIFGEKQSGFLSEMRENYEYAFDYYYG-VSGSYEADDISHWYRSPVELGWGNRIKFDH 298
BrdA1[BD3100]      200 YEVHGVIEDAHAVHSALVAAGGPFGLERLGMQVYGMNHTENGFPQAHIHFLSAWLQD--PAFIAYLGDAWDSWYACSFTDFAGSAGDD-IEKRYCNPVELGWGHVVKFDH 306
BrdA2[BD3100]      194 YEVHGRIEDAEAIYSALRAAGEPFGMERLGMQVYGMNHTEGGFAQSYIHFLPAYTQD--AAFMDYLQGEADAFMS----DLPGSAGPD-IEKRYANPVELGWGHMIRFDH 296
                        *.         .  ..  **    *.   * .      * .           . *         .                  **     .   * * * * *   . ***

BrdA[BD7100]       296 KFRGDEALRVEKADPKRTLCTLKWNSDDIIDIYASLFRQGD-LPDFMEMPQDPRH-----YLYFDKILKDG-KEVGATSSRGYSAHFREMISLAVLDIELAVPGTDVTVV 398
BrdA[GBD-1]        297 EFIGADALREEVASPRRSGATLVWNSEDIIDLYASLFRKGEPLPDFMELPQDPRG-----YVYADSVLKNG-ERVGITSSRGYSAYFREMISLCVLDVEHNVPGDEVTVL 400
CDS4435[GBD-1]     320 DFIGREALEQIDPATQRKKITLAWDTDDLAKIFASMVSPEGIGYKYFDLPLANYG-----YFNFDSVLDAGGTNVGLSMWTGYSANERRALSLATVDPS-IEIGTELKVV 423
BrdA1[CJ1]         299 DFIGAEALRKELAEPRRRIATLVWNSEDVVDLYASFFRKGEPLPDFMELPRDPRG-----YVYADKVMKDG-KLVGMTTSRGYSAYFREMISLCVIDLDQHAPGTQVTVI 402
BrdA2[CJ1]         299 DFIGSEALRKELAEPKRRIASLVWNSEDIIALYGDLFRKGEPLPDFMEMPQDPRG-----YMHSDKVMKNG-KIIGMTSSRGYSAYFREVISLCMIDLEHHTPGTEVTVV 402
BrdA1[BD3100]      307 DFIGRAALEAEMAAPRRKMVTLIWNAEDVLDVYASHLRPGE-EYAFMDFLSNPIWNRLGMGSHVDDVLKGD-EAVGVSMGRIYSYYYRAMISLCTIDLPHGEIDNEVEIV 414
BrdA2[BD3100]      297 DFTGRAALERIMAGSHRRIVTLEWDQDAILDVYASQFRADA-DIEFMDFAANPVWTAHNSVVFSDDVFVGD-TLVGISSGRVFSYYYRKMLSLAVLDPGHAEIGREVEVL 404
                        * *  **       .*   .* *  . .  ..          . .                 * .       .* .    .*  *  .**. .*            . ..

BrdA[BD7100]       399 WGAPG----------SPQREIRATTALAPYKEV--RSRVDLTTLPARPF------------- 435
BrdA[GBD-1]        401 WGNPG----------TAQREIRATIAPAPYKPN--RSRVDLHSL------------------ 432
CDS4435[GBD-1]     424 WGEPNGGSEKATVEPHEQFEVKAIASPVPYTAVARENYQSGWRTKVLA-------------- 471
BrdA1[CJ1]         403 WGNPG----------TPQREIRATVAPAPYKTD--RGRVDFATLPSYR-------------- 438
BrdA2[CJ1]         403 WGNPG----------TPQREIRATVAPAPYKKD--RARVDFATLPSHLGK------------ 440
BrdA1[BD3100]      415 WGAPG----------TRQKKIRATVARFPYLDLPRNETIDVNTIPRLPAASEAELSPAS--- 463
BrdA2[BD3100]      405 WGSPD----------NRQKRIKAKVARFPYLDLPKNADIDVRSLDGAFAGKEAPSDQTVTLP 456
                       ** *            *  ..*    .  **
```

Supplementary Figure 6. Amino acid sequence alignment of predicted BrdA found in the BBR-degraders.
Amino acid sequences of BrdA found in the genome sequences of BD3100, GBD-1 and CJ1 were aligned by ClustalW (v1.83) multiple sequence alignment. Identical amino acids are indicated by asterisks. Similar amino acids are indicated by dots.

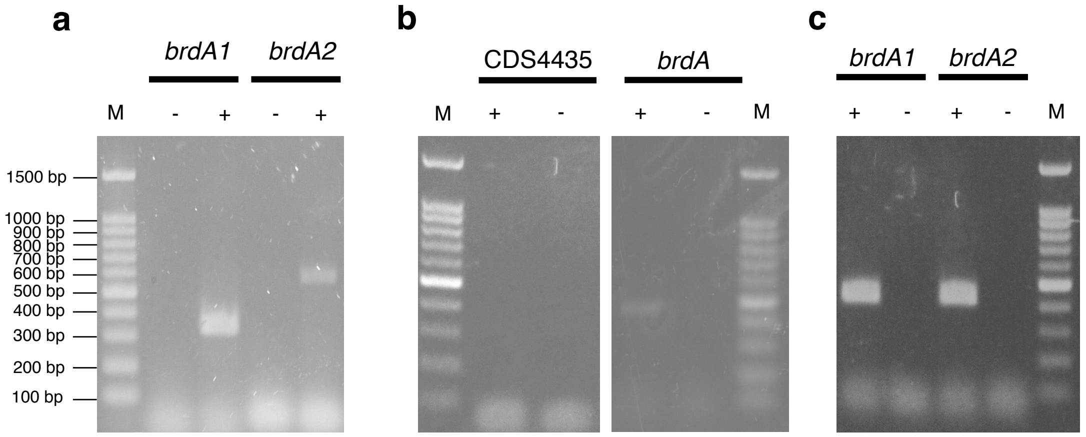

Supplementary Figure 7. Transcription of *brdA* homologues in BBR-utilizing bacteria.
(a) RT-PCR analysis of *brdA1* and *brdA2* genes in *Sphingobium* sp. BD3100. (b) RT-PCR analysis of CDS4435 and *brdA* in *Arthrobacter* sp. GBD-1. (c) RT-PCR analysis of *brdA1* and *brdA2* genes in *Burkholderia* sp. CJ1. Total RNA from cells grown in LB containing 0.5 mM BBR was reverse-transcribed. RNA samples were concurrently analysed in PCR mixtures with (+) and without (-) reverse transcriptase (RT) to verify the absence of total DNA. Primer sets listed in Supplementaly Table 4 were designed to amplify cDNAs in each internal region of each *brdA* homologue. The lane of the DNA ladder marker is indicated by M. The deduced sizes of RT-PCR products of *brdA1* and *brdA2* of BD3100 were 363 and 582 bp, respectively. The deduced sizes of RT-PCR products of CDS4435 and *brdA* of GBD-1 were 428 and 475 bp, respectively. The deduced sizes of RT-PCR products of *brdA1* and *brdA2* of CJ1 were 424 and 469 bp, respectively.

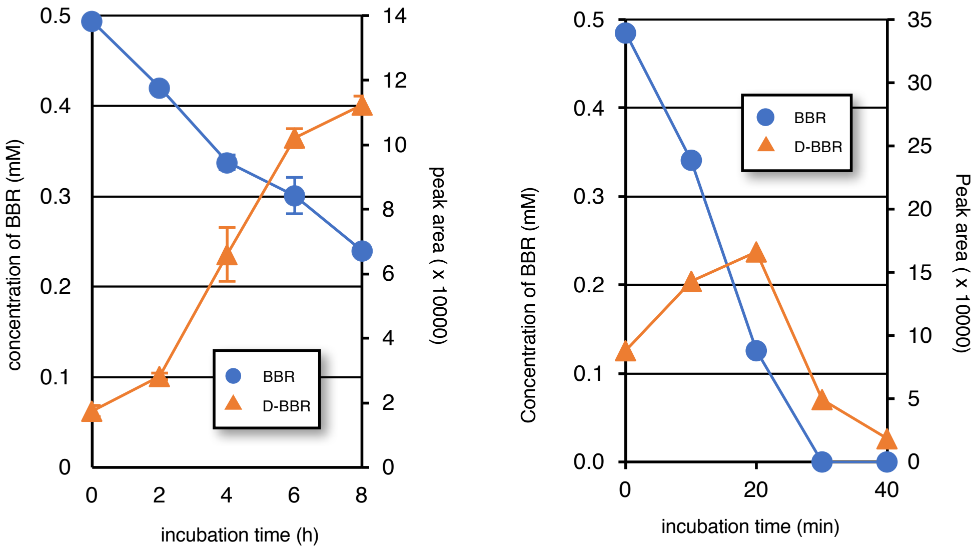

Supplementary Figure 8. Degradation ability of BBR in BDΔ*brdA1* and BDΔ*brdA2*.
Time-course analysis of BBR concentration and peak area of D-BBR in the resting-cell assay of BDΔ*brdA1* (left panel) and BDΔ*brdA2* (right panel). Circles indicate concentrations of BBR. Triangles indicate peak areas of D-BBR. Each value is the average of at least three measurements. The vertical lines indicate the standard deviations from the means. Both mutants converted BBR to D-BBR. These results suggest that both *brdA1* and *brdA2* are involved in BBR degradation. BDΔ*brdA2* degrades BBR better than BDΔ*brdA1*.

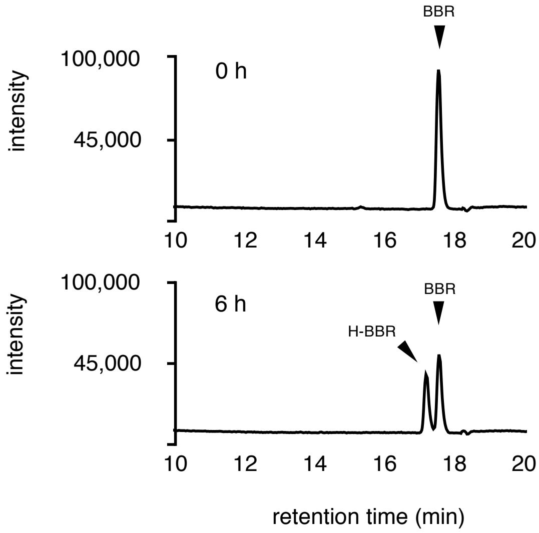

Supplementary Figure 9. BDΔ*brdA12* lost the ability to demethylenate.

HPLC chromatograms monitored at 280 nm. The resting-cell assay after reaction times of 0 (upper) and 6 h (bottom). Peaks of BBR and H-BBR are indicated by arrowheads. The peak at a retention time of 17.2 min corresponds to H-BBR. Accumulation of H-BBR is observed in BDΔ*brdA12*. The demethylenation products, D-BBR and HD-BBR (see Fig. 1), were not observed in BDΔ*brdA12*. This result suggests that both *brdA1* and *brdA2* are responsible for the demethylenation of BBR and H-BBR.

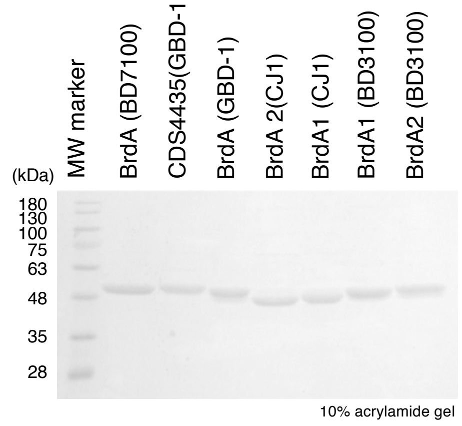

Supplementary Figure 10. Purification of His-BrdA homologues.
His-BrdA homologues, which are expressed in *E. coli* BL21 (DE3), were purified by HisPure Ni-NTA resin (Thermo). Three hundred ng of each purified protein was analysed by SDS-PAGE. Proteins were detected by CBB stain.

```
BrdA[BD7100]       1 -----MPSFRDSLVAQLRHPIQFHAGDMWGPPQYTNWMDEERSWKDSCYLGDWTFLP---TIRYTGPDVLKLFADCSVNT  72
BrdA2[CJ1]         1 --MKTENSTPNLSMASFRRPIRPHFGDAWGPPQFTNWIDESLSWKETCYLGDWTFLP---ALKYTGPDVLKLFADTSVNT  75
BrdA1[CJ1]         1 --MENSHQKLDFSVKSLRRPLKSHFTDSWGPPQFTNWVDESMSWKETCYLGDWTFLD---ALKYTGPDVLKLFADTSVNT  75
BrdA[GBD-1]        1 ---MTQTPSPQFSVSALRNPIREHHGAVWGPPQYTNWIEESLSWKETCYLGDWTFLP---SMRYTGPDVLRLFSDVSVNT  74
BrdA2[BD3100]      1 -----MIPHSPYLPFDPDVSDYNVAFTHLSPWEFGGWKRESLSWKKGCYLHAGLNPPS--PYRLSGPDALALLRDACING  73
BrdA1[BD3100]      1 MTDPTLLPHSPYLPYDQSLLNYNMTYGHLDPMEFGGWKRETLSWKEGCYLHAGLNPPS--PYRISGPDAIRLFSDACVNS  78
SesA[no.22]        1 ---------MTAEQAINEGAFSLAASFGFVPLEYRGYEAEVLASKETAYIGTALNGAMSPIYDVTGPDALEFLRSVCINS  71
                                                 + **+..+.+ *  . *  .*.+++++++      +.***+. ++ + ..*+

BrdA[BD7100]      73 MNNFKIGQSKHIIHTNRDGKVIEDGLLTRTADEELICYSSY-WADYIRRNGNYRVD--MEPIEQVKFHLQGPNALFVLET 149
BrdA2[CJ1]        76 MENFAIGQSKHVIHCNEDGKIIEEGILSRFSENEYVAFSMY-WADYVRRQGNYDVEP-AELLPLTKHHLQGPNALFVLEK 153
BrdA1[CJ1]        76 MQNFEIGQSKHVIHCNEDGKIIEEGILSRFGENEYVAFSMY-WADHVRRQGNYDVNP-PELLPLTKFHLQGPNALFVLEK 153
BrdA[GBD-1]       75 MNNFAIGQSKHIIQCDENGKIIDDAVLSRTGEQEVISFSTF-WADYVRRQGDYDVE--AEYVGLSKFHLQGPTSLFVLES 151
BrdA2[BD3100]     74 FSTFSIGCSKHAVMCNAQGNVMADGMVLRTGEEDFTCFFLNPYIDYLAASGRYDVRGEDLSGKVFLFQVAGPRSLEVMEA 153
BrdA1[BD3100]     79 FAKFSIGGSKHAIMCNAEGNIMAHGMLLRTGEEEFQSFFLSPYINYLVESGKYDVRGEDMTGKVFMFQVAGPRSLEVIEA 158
SesA[no.22]       72 FRGFQVGQIRHAVLCNDKGQILTDGVVARIDEDTYRTYWLAPALEYRLINSGLDVKGEDQSSNEFFFQLAGPRSLEVLEA 151
                        * .*++.* . .   *+..   .. *  . +  ..+   +++  ++ + + *    +     + ..+** .*+*.*

BrdA[BD7100]     150 ALGRDFRDLKFMRNEDVTIA-----GVPTRVLRQGMSGEIGFELQADKAQGQILRETILEAGTKYGIHEMGGRVAMLNHL 224
BrdA2[CJ1]       154 VANESLRDLKFMRFRKIRIA-----GHEVLALRQGMSGEIGFELQGPLEHREEIWNTILEAGREFGIRQMGGRVAMINHL 228
BrdA1[CJ1]       154 VANESLRDLKFMRFRKIQIA-----GHEVIALRQGMSGEIGFELQGPLEHREEIWNAIFEAGQEFGIRQMGGRVAMINHL 228
BrdA[GBD-1]      152 AAQASVRDLKFMRSMKVSIA-----DVEVTVLRQGMSGEVGFELQFPIEKADVVRAALLEAGSDHGMKQMGGRVAMLNHL 226
BrdA2[BD3100]    154 ATGENLRDIEFLWHRASTIR-ADGRDVPVRVFRLGVARTLAYEVHGRIEDAEAIYSALRAAGEPFGMERLGMQVYGMNHT 232
BrdA1[BD3100]    159 ATQEDLHDIKFLRHRPSQIVGADGRKIQVRIVRIGMAGTLAYEVHGVIEDAHAVHSALVAAGGPFGLERLGMQVYGMNHT 238
SesA[no.22]      152 AAHEDLHDIAFGRHRMSTIA-----GIPVRILRLGMAGGLAYEVHGAAADTETAYRAIWEAGQPFGLVKQGLNAYLMQHT 226
                           .*.+*++     *+           +*+*..++.+.*..           .. +**   *.   *+++++..*+

BrdA[BD7100]     225 QAAYPTVMTDYLPAMYDDDGAGYLEEYMGEADGYFARYYGAVAGSFESDDVSGWYRSPVELGWGGRINFDHKFRGDEALR 304
BrdA2[CJ1]       229 EANYPTICLDYMPAIFGEKQSGFLSEMRENYEYAFDYYYG-VSGSYEADDISHWYRSPVELGWGNRIKFDHDFIGSEALR 307
BrdA1[CJ1]       229 EANYPTNALDYLPAIFDENNSAYLGEMFTNYKELFDYYFR-VAGSYDSSSVADWYRSPVELGWGNRIKFDHDFIGAEALR 307
BrdA[GBD-1]      227 EAYYPTQGLDYMPAVFDGRHPDFLHELVENGGGWMDIYYR-VAGSFESDDIRDWYRSPVEFGWGNRINFDHEFIGADALR 305
BrdA2[BD3100]    233 EGGFAQSYIHFLPAYTQD--AAFMDYLQGEADAFMS----DLPGSAGPD-IEKRYANPVELGWGHMIRFDHDFTGRAALE 305
BrdA1[BD3100]    239 ENGFPQAHIHFLSAWLQD--PAFIAYLGDAWDSWYACSFTDFAGSAGDD-IEKRYCNPVELGWGHVVKFDHDFIGRAALE 315
SesA[no.22]      227 EAGFPNINLHYPLPWYED--PDMAAFFDTRPTQNFYNKYRFFYGSVGPD-AEARFVTPYQIGLGKMVDFNHDFIGKEALQ 303
                     .  .++   +. ++           +           +   + **        +.++*+. *+*  . *+* * *  **+

BrdA[BD7100]     305 VEKADPKRTLCTLKWNSDDIIDIYASLFRQGD-LPDFMEMPQDPRH----------YLYFDKILKDGKEVGATSSRGYSA 373
BrdA2[CJ1]       308 KELAEPKRRIASLVWNSEDIIALYGDLFRKGEPLPDFMEMPQDPRG----------YMHSDKVMKNGKIIGMTSSRGYSA 377
BrdA1[CJ1]       308 KELAEPRRRIATLVWNSEDVVDLYASFFRKGEPLPDFMELPRDPRG----------YVYADKVMKDGKLVGMTTSRGYSA 377
BrdA[GBD-1]      306 EEVASPRRSGATLVWNSEDIIDLYASLFRKGEPLPDFMELPQDPRG----------YVYADSVLKNGERVGITSSRGYSA 375
BrdA2[BD3100]    306 RIMAGSHRRIVTLEWDQDAILDVYASQFRADA-DIEFMDFAANPVWTAH-----NSVVFSDDVFVGDTLVGISSGRVFSY 379
BrdA1[BD3100]    316 AEMAAPRRKMVTLIWNAEDVLDVYASHLRPGE-EYAFMDFLSNPIWNRL-----GMGSHVDDVLKGDEAVGVSMGRIYSY 389
SesA[no.22]      304 REAEADHWAAVTLVWNEDDVADVVASKYRGRD-VEPYDKIDDRPFDIYHNLGQPGFAYHADWVLADGERIGTSTGRINSV 382
                      + + +.+   .* *++.+.  .+   +* +  +++.++ + +*+           +   * . + +  .* . +*++*+

BrdA[BD7100]     374 HFREMISLAVLDIELAVPGTDVTVVWGAPGSPQREIRATTALAPYKEV--RSRVDLTTLPARPF------------- 435
BrdA2[CJ1]       378 YFREVISLCMIDLEHHTPGTEVTVVWGNPGTPQREIRATVAPAPYKKD--RARVDFATLPSHLGK------------ 440
BrdA1[CJ1]       378 YFREMISLCVIDLDQHAPGTQVTVIWGNPGTPQREIRATVAPAPYKTD--RGRVDFATLPSYR-------------- 438
BrdA[GBD-1]      376 YFREMISLCVLDVEHNVPGDEVTVLWGNPGTAQREIRATIAPAPYKPN--RSRVDLHSL------------------ 432
BrdA2[BD3100]    380 YYRKMLSLAVLDPGHAEIGREVEVLWGSPDNRQKRIKAKVARFPYLDLPKNADIDVRSLDGAFAGKEAPSDQTVTLP 456
BrdA1[BD3100]    390 YYRAMISLCTIDLPHGEIDNEVEIVWGAPGTRQKKIRATVARFPYLDLPRNETIDVNTIPRLPAASEAELSPAS--- 463
SesA[no.22]      383 YYRRMISLGFIDKRHAAEGTELTVLWGRPGTPQKEIRVTVGRYPYFDLEKNNAIDVASIPRPALDVSAGA------- 452
                      .*+..**  .*     ++  .+..** *+  *.+*.++ + +***+   + +.*  ..
```

Supplementary Figure 11. Amino acid sequence alignment of BrdA and SesA.

Identical amino acids are indicated by asterisks. Identical amino acids of BrdA [BD7100], BrdA1 [CJ1], BrdA2 [CJ1] and BrdA [GBD-1] are indicated by plus signs. Red and green characters indicate predicted proton donors for the demethylenation of methylenedioxy ring and residues predicted in SesA to hydrogen bond to THF, respectively (Kumano et al, PNAS 2016 vol. 113, pp. 9087–9092) .

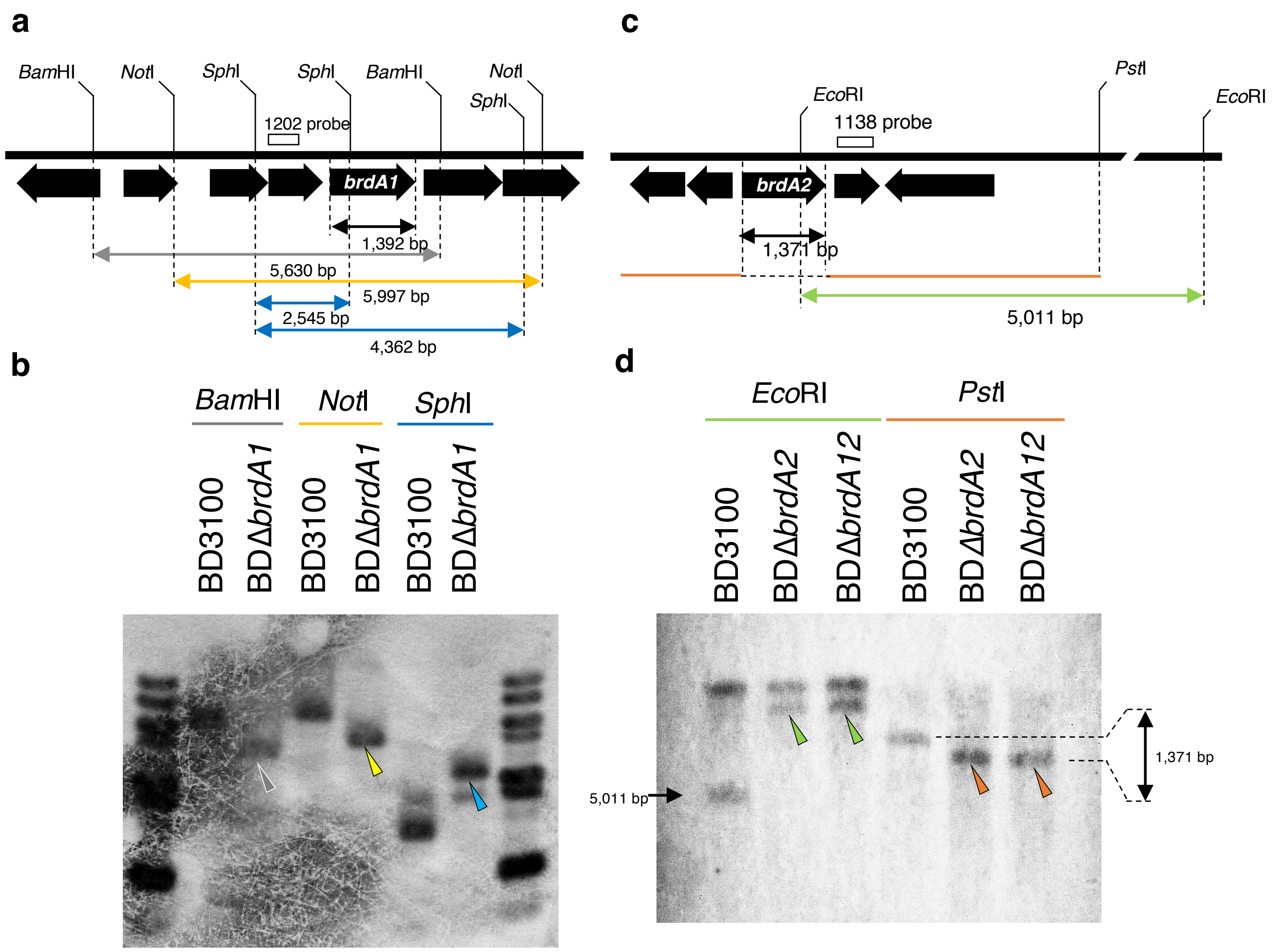

Supplementary Figure 12. Southern hybridization analysis of deletion mutants of BD3100.
The deletion mutants *brdA1*, *brdA2* and both *brdA1* and *brdA2* are designated BDΔ*brdA1*, BDΔ*brdA2* and BDΔ*brdA12*, respectively. The gene deletion was confirmed by Southern hybridization using 1202 and 1138 probes, which are indicated by open boxes. (a) ORF and restriction map of the *brdA1* region. The black double-headed arrow shows the size of *brdA1*. Grey, yellow and blue double-headed arrows show the sizes of *Bam*HI, *Not*I and *Sph*I DNA fragments, respectively. (b) Southern hybridization analysis of BDΔ*brdA1* using the 1202 probe. Grey and yellow arrowheads indicate hybridization bands, which are down-shifted in BDΔ*brdA1*. The blue arrowhead indicates the hybridization band, which is up-shifted in BDΔ*brdA1* because of the lack of a *brdA1* gene with *Sph*I site. (c) ORF and restriction map of the *brdA2* region. The black double-headed arrow shows the size of the *brdA2* gene. The orange lines indicate the *Pst*I DNA fragment of BDΔ*brdA2* and BDΔ*brdA12*, produced by deletion of the *brdA2* gene. The green blue double-headed arrows show the size of the *Eco*RI DNA fragments. (d) Southern hybridization analysis of BDΔ*brdA2* and BDΔ*brdA12* using the 1138 probe. The green arrowheads indicate the hybridization bands, which are up-shifted in BDΔ1137 and BDΔ*brdA*. The orange arrowheads indicate the hybridization bands, which are down-shifted in BDΔ*brdA2* and BDΔ*brdA12* because of the lack of *brdA2* in the *Eco*RI site.

Supplementary Table 2. Identity and similarity matrix of BrdA homologues in BBR degraders.

	BrdA [BD7100]	BrdA [GBD-1]	BrdA1 [CJ1]	BrdA2 [CJ1]	BrdA1 [BD3100]	BrdA2 [BD3100]	CDS4435 [GBD-1]
							Identity scores (%)
BrdA [BD7100]	100.0	60.0	59.6	58.6	30.5	30.3	28.8
BrdA [GBD-1]	75.6	100.0	65.0	63.9	31.8	35.1	28.0
BrdA1 [CJ1]	76.0	80.7	100.0	80.2	28.9	32.8	30.1
BrdA2 [CJ1]	74.5	79.5	89.8	100.0	29.3	33.8	28.5
BrdA1 [BD3100]	46.4	47.6	47.3	47.5	100.0	60.9	23.4
BrdA2 [BD3100]	48.5	51.2	51.4	50.7	75.1	100.0	26.6
CDS4435 [GBD-1]	44.9	44.1	46.0	46.2	38.7	41.2	100.0
	Similarity scores (%)						

Supplementary Tables

Supplementary Table 1. Identity and similarity of the amino acid sequence of CDS6194

Accession No.	Classification	Organism	Identity (%)	Similarity (%)
WP_010952964.1	glycine cleavage system protein T	*Pseudomonas putida*	50	70
WP_037481219.1	glycine cleavage system protein T	*Sphingomonas paucimobilis*	50	68
WP_030540999.1	glycine cleavage system protein T	*Sphingobium* sp. DC-2	50	68
WP_048466571.1	glycine cleavage system protein T	*Methylobacterium aquaticum*	50	69
WP_008330104.1	glycine cleavage system protein T	*Maritimibacter alkaliphilus*	49	69
WP_035689777.1	glycine cleavage system protein T	*Bradyrhizobium elkanii*	48	69
WP_028142991.1	glycine cleavage system protein T	*Bradyrhizobium* (Multispecies)	48	68
WP_026201551.1	glycine cleavage system protein T	*Bradyrhizobium* sp. WSM2793	48	68
WP_038971509.1	glycine cleavage system protein T	*Bradyrhizobium* sp. CCBAU 15635	48	68
WP_038947857.1	glycine cleavage system protein T	*Bradyrhizobium* sp. CCBAU 15544	48	68

Supplementary Table 3. Strains and plasmids used in this study.

Strain or plasmid	Relevant characterisitic(s)[a]	Reference or source
Rhodococcus sp.		
BD7100	Wild type, BBR degrader	Takada, H. et al., 2015
TA140	A transposome mutant of BD7100, Ts^r	This study
Sphingobium sp.		
BD3100	Wild type, BBR degrader	Takada, H. et al., 2015
BDΔ*brdA1*	Deletion mutant of *brdA1*	This study
BDΔ*brdA2*	Deletion mutant of *brdA2*	This study
BDΔ*brdA12*	Deletion mutant of *brdA1* and *brdA2*	This study
Arthrobacter sp.		
GBD-1	Wild type, BBR degrader	This study
Burkholderia sp.		
CJ1	Wild type, BBR degrader	This study
Escherichia coli		
DH5α	F$^-$, Φ80d*lacZ*ΔM15, Δ(*lacZYA-argF*)U169, *deoR*, *recA*1, *endA*1, *hsdR*17(r_K^-, m_K^+), *phoA*, *supE*44, λ$^-$, *thi*-1, *gyrA*96, *relA*1	TAKARA BIO Inc. (Shiga, Japan)
BL21 (DE3)	*fhuA*2 [lon] *ompT* gal (λ DE3) [dcm] Δ*hsdS*, λ DE3 = λ sBamHIo ΔEcoRI-B int::(*lacI*::*PlacUV*5::T7 gene1) i21 Δ*nin*5	TAKARA BIO Inc. (Shiga, Japan)
Plasmids		
pUC19	Cloning vector; Amp^r	TAKARA BIO Inc. (Shiga, Japan)
pU*brdA*T	pUC19 with 1.4-kb NcoI and *Hin*dIII fragment carrying *brdA*	This study
pU1201	pUC19 with 1.3-kb PCR fragment carrying *CDS1201*	This study
pU1137	pUC19 with 1.3-kb PCR fragment carrying *CDS1137*	This study
pU4430	pUC19 with 1.3-kb PCR fragment carrying *CDS4430*	This study
pU4435	pUC19 with 1.3-kb PCR fragment carrying *CDS4435*	This study
pU7326	pUC19 with 1.3-kb PCR fragment carrying *CDS7326*	This study
pU7349	pUC19 with 1.3-kb PCR fragment carrying *CDS7349*	This study
pTip-QC1	Expression vector; Ts^r Cm^r	Nakashima, N. and Tamura, T., 2004
pTQ*brdA*	pTip-QC1 with 1.4-kb *Nco*I and *Hin*dIII fragment of pU*brdA*T	This study
pET-28a(+)	Expression vector; Km^r T7 promoter	Merck KGaA (Darmstadt, Germany)
pE*brdA*	pET-28a(+) with 1.3-kb *Nde*I and *Hin*dIII fragment of pU*brdA*E	This study
pE1201	pET-28a(+) with 1.3-kb *Bam*HI and *Hin*dIII fragment of pU1201	This study
pE1137	pET-28a(+) with 1.3-kb *Bam*HI and *Hin*dIII fragment of pU1137	This study
pE4430	pET-28a(+) with 1.3-kb *Eco*RI and *Hin*dIII fragment of pU4430	This study
pE4435	pET-28a(+) with 1.3-kb *Nde*I and *Hin*dIII fragment of pU4435	This study
pE7326	pET-28a(+) with 1.3-kb *Nde*I and *Hin*dIII fragment of pU7326	This study
pE7349	pET-28a(+) with 1.3-kb *Nde*I and *Hin*dIII fragment of pU7349	This study
pTNR-TA	Transposon vector; Ts^r	Sallam, K. I. et. al., 2006
pK18mobsacB	*oriT sacB* Km^r	National BioResource Project (NIG,Japan)
pU1201UP	pUC19 with PCR fragment carrying upstream of *brdA1* of BD3100	This study
pU1201DOWN	pUC19 with PCR fragment carrying downstream of *brdA1* of BD3100	This study
pK18-1201UP	pK18*mobsacB* with 0.9-kb *Eco*RI and *Xba*I fragment of pU1201UP	This study
pK18Δ1201	pK18-1201UP with 0.9-kb *Xba*I and *Hin*dIII fragment of pU1201DOWN	This study
pU1137UP	pUC19 with PCR fragment carrying upstream of *brdA2* of BD3100	This study
pU11137DOWN	pUC19 with PCR fragment carrying downstream of *brdA2* of BD3100	This study
pK18-1137UP	pK18*mobsacB* with 1.1-kb *Eco*RI and *Xba*I fragment of pU1137UP	This study
pK18Δ1137	pK18-1137UP with 0.9-kb *Xba*I and *Hin*dIII fragment of pU1137DOWN	This study

[a] Abbreviations: Amp^r, Km^r, Cm^r and Ts^r, resistance to ampicillin, kanamycin, chloramphenicol and thiostrepton, respectively

Supplementary Table 4. Oligonucleotides used in this study.

Name	Sequence* (5'-3')	Use
TNR-TAinvF	ACGAGAGGATCGACAGGAATCTCG	Inverse PCR
TNR-TAinvR	ATTTGCGATGGTGTCCAACTCAGTC	Inverse PCR, Sequencing
ETNde6194Fw	CATATGCCCTCTTTCCGCGATTCACTTGTC	In fusion cloning for pET-28a(+)
ETHin6194Rv	AAGCTTTCAGAATGGCCGAGCGGGTAACG	In fusion cloning for pET-28a(+)
CDS6194NcoFw	CCATGGGCGGCCGAAGGAACCAG	Cloning for pTip-QC1
CDS6194HinRv	AAGCTTTCAGAATGGCCGAGCGGGTAAC	Cloning for pTip-QC1
27F	GAGTTTGATCCTGGCTCAG	16S rRNA
1525R	GAGGTGATCCAGCCGCAGG	16S rRNA
M13-20	CGACGTTGTAAAACGACGGCCAGT	Sequencing
Rv-M	GAGCGGATAACAATTTCACACAGG	Sequencing
RT6194Fw	CAGTACACCAACTGGATGGATGAGGAG	RT-PCR of *brdA* gene from BD7100
RT6194Rv	TCAGAATTCCACTTCAGAGTGCACAGAG	RT-PCR of *brdA* gene from BD7100
RT1201Fw	ATGGCATGAACCATACGGAAAACG	RT-PCR of *brdA1* gene from BD3100
RT1201Rv	AGTCCATGAACGCATATTCCTCGC	RT-PCR of *brdA1* gene from BD3100
RT1137Fw	CAAGGTGTTCCTGTTCCAGGTAGCC	RT-PCR of *brdA2* gene from BD3100
RT1137Rv	TAGACATCGAGAATGGCGTCCTGATCC	RT-PCR of *brdA2* gene from BD3100
RT4435Fw	ATACCGTGAATGGCTTGGCG	RT-PCR of CDS4435 gene from GBD-1
RT4435Rv	CGATTTCAATCGACGGATCG	RT-PCR of CDS4435 gene from GBD-1
RT4330Fw	GGCATGAAGCAAATGGGAGG	RT-PCR of *brdA* gene from GBD-1
RT4330Rv	AACTTGTGATTCCCACCCGC	RT-PCR of *brdA* gene from GBD-1
RT7326Fw	GAATGCCATTTTCGAAGCGG	RT-PCR of *brdA1* gene from CJ1
RT7326Rv	GCAACTCCATGAAATCGGGC	RT-PCR of *brdA1* gene from CJ1
RT7349Fw	CTTCTCGCCAAATATCGCCG	RT-PCR of *brdA2* gene from CJ1
RT7349Rv	CGAAGACGGCAAGATCATCG	RT-PCR of *brdA2* gene from CJ1
1202probeFw	CACAACAAGGCAGACTGATCGACG	Probe for Southern hybridization
1202probeRv	ATGAGTCACCGCCTCATCGAAGG	Probe for Southern hybridization
1138probeFw	GGTGACACGCTGGTGATATGGAAGC	Probe for Southern hybridization
1138probeRv	GTAGAAGGTAGAACGGCCGACCTGTAGC	Probe for Southern hybridization
ETBam1201Fw	GGATCCATGACTGATCCCACGCTCCTCCC	Cloning of *brdA1* gene from BD3100
ETHin1201Rv	AAGCTTGCGACCGATCAGCTCGCCG	Cloning of *brdA1* gene from BD3100
ETBam1137Fw	GGATCCATGATCCCCCACAGCCCCTATCTG	Cloning of *brdA2* gene from BD3100
ETHin1137Rv	AAGCTTTTATGGCAACGTCACCGTTTGGTC	Cloning of *brdA2* gene from BD3100
ETEco4430Fw	AAATGACACAGACTCCGTCGCCGCAG	Cloning of *brdA* gene from GBD-1
ETHin4430Rv	TCAGAGCGAATGGAGATCGACACGAC	Cloning of *brdA* gene from GBD-1
ETNde4435Fw	CATATGGTGAATGTGAATAAGCCAAAAAGC	Cloning of *CDS4335* gene from GBD-1
ETHin4435Rv	CTACGCAAGAACCTTGGTGCGCCAAC	Cloning of *CDS4335* gene from GBD-1
ETNde7326Fw	CATATGGAAAACTCTCACCAAAAACTAGAT	Cloning of *brdA1* gene from CJ1
ETHin7326Rv	TCAGCGGTATGACGGGAG	Cloning of *brdA1* gene from CJ1
ETNde7349Fw	CATATGGCGTCGTTTCGCCG	Cloning of *brdA2* gene from CJ1
ETHin7349Rv	CTACTTCCCGAGGTGCGAGGGCAG	Cloning of *brdA2* gene from CJ1
1201UPFw	GGATCCAGCGCTGTCTATGTCCGGTCG	Construction of plasmid for deletion mutant of *brdA1* gene in BD3100
1201UPRv	TCTAGACATATGCGAACTCCGTCGATTGC	Construction of plasmid for deletion mutant of *brdA1* gene in BD3100
1201DOWNFw	TCTAGATGATCGGTCGCGACGCCGTTCTGC	Construction of plasmid for deletion mutant of *brdA1* gene in BD3100
1201DOWNRv	AAGCTTCTGACACATCCTCGAACCAAGCG	Construction of plasmid for deletion mutant of *brdA1* gene in BD3100
1137UPFw	GGATCCGCGCCTCTGGTGACTGGTACGG	Construction of plasmid for deletion mutant of *brdA2* gene in BD3100
1137UPRv	TCTAGAGATCATTTTGGTATCGCTCACGCG	Construction of plasmid for deletion mutant of *brdA2* gene in BD3100
1137DOWNFw	TCTAGACCATAAAGGAACCTCCAACGGGAC	Construction of plasmid for deletion mutant of *brdA2* gene in BD3100
1137DOWNRv	AAGCTTGTGTTTGCCGATGCCAAGGGTAAG	Construction of plasmid for deletion mutant of *brdA2* gene in BD3100

*, Addition of restriction sites in the oligonucleotides were indicated by underlines.
